# Involvement of the ubiquitin-proteasome system in the regulation of the tumor microenvironment and progression

**DOI:** 10.1016/j.gendis.2024.101240

**Published:** 2024-02-02

**Authors:** Yulan Huang, Yuan Gao, Zhenghong Lin, Hongming Miao

**Affiliations:** a Department of Pathophysiology, College of High Altitude Military Medicine, Army Medical University, Chongqing 400038, China; b School of Life Sciences, Chongqing University, Chongqing 401331, China; c Jinfeng Laboratory, Chongqing 401329, China

**Keywords:** Adipose cells, Deubiquitination, Immunity, Tumor microenvironment, Tumor-associated fibroblasts, Ubiquitination

## Abstract

The tumor microenvironment is a complex environment comprising tumor cells, non-tumor cells, and other critical non-cellular components. Some studies about tumor microenvironment have recently achieved remarkable progress in tumor treatment. As a substantial part of post-translational protein modification, ubiquitination is a crucial player in maintaining protein stability in cell signaling, cell growth, and a series of cellular life activities, which are also essential for regulating tumor cells or other non-tumor cells in the tumor microenvironment. This review focuses on the role and function of ubiquitination and deubiquitination modification in the tumor microenvironment while discussing the prospect of developing inhibitors targeting ubiquity-related enzymes, thereby providing ideas for future research in cancer therapy.

## Introduction

**Figure 1:**
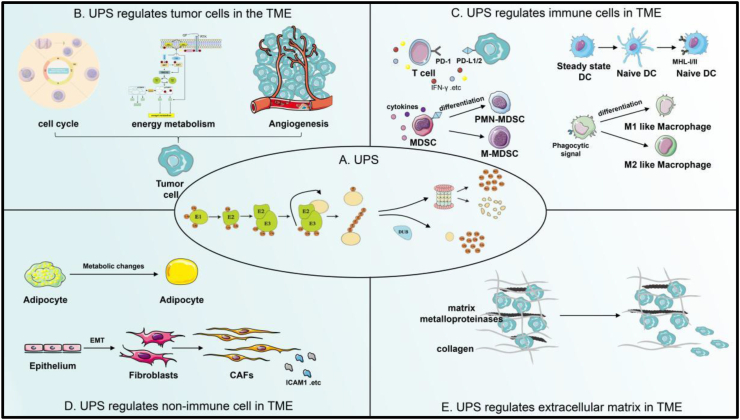
The UPS regulates the TME. **(A)** E1 ubiquitin-activating enzyme activates the carboxyl group of the C terminus of ubiquitin in an ATP-dependent manner through the formation of high-energy thioester bonds. Next, the ubiquitin molecule, which binds to E2, is moved to the targeting protein with the help of E3 ligase. Then, substrate protein labeled with ubiquitin enters into 26s proteasome for degradation, broken down into polypeptides and small-molecule amino acids. Deubiquitinase is to avoid degradation of substrate proteins by removing the ubiquitin tag from the substrate proteins. **(B)** UPS regulates tumor cells in the TME by regulating the levels of proteins related to the cell cycle, energy metabolism, and angiogenesis. **(C)** UPS regulates the anti-tumor immunity of T cells by regulating the protein levels of PD-1/PD-L1 and some inflammation-related cytokines. It also regulates the anti-tumor immunity of immune cells in the TME by regulating the maturation and anti-inflammatory presentation ability of DCs, the differentiation and their cytokines secretion of MDSCs, and the polarization of macrophages. **(D)** UPS modulates their role in tumor progression by regulating the lipid metabolism of adipocytes and the transformation and protein formation of CAFs. **(E)** UPS regulates tumor progression by regulating the levels of metalloproteinases and collagen in the TME. UPS, ubiquitin-proteasome system; TME, tumor microenvironment; PD-1/PD-L1, programmed death-1/ligand-1; DC, dendritic cell; PMN/M-MDSC, polymorphonuclear/monocytic-myeloid-derived suppressor cells; CAFs, cancer-associated fibroblasts; ICAM1, intercellular adhesion molecule 1.

Tumors are formed because of many reasons. For example, when cells in the cell cycle lose their regulation, control of cell proliferation is lost. Under normal circumstances, such cells are eliminated by the immune recognition function or may escape from immune cell monitoring by changing their surface antigens. Tumor cells compete with each other and eventually develop into malignant tumors, leading to cancer occurrence.^1^^,^^2^ In addition to changing their surface antigen as mentioned above, tumor cells can affect immune cells or tissue components, thereby creating an environment conducive to tumor growth.^3^ Such an environment with hypoxia,^4^ poor nutrients,^5^ high acidity,^6^ and an immunosuppressive microenvironment^7^ contains tumor cells as well as immune cells such as T cells, dendritic cells (DCs), macrophages, myeloid-derived suppressor cells (MDSCs), and regulatory T cells (Fig. 1). Other non-immune cells and cytokines also occupy crucial positions in the tumor microenvironment (TME).^8^^,^^9^

Being a part of post-translational protein modification, ubiquitination is closely related to various physiological cellular activities, including regulation of protein transcription and interactions, DNA replication, cell growth response, immune responses, and signal transduction.^10^, ^11^, ^12^ Ubiquitin modification is a reversible enzymatic cascade wherein ubiquitin ligases and deubiquitinating enzymes precisely regulate substrates. The ubiquitin molecule is a 76-amino acid-long protein, where adjacent amino acids directly form proteins through covalent binding. This molecule includes seven lysines (K6, K11, K27, K29, K33, K48, and K63).^13^

Ubiquitin is modified through monoubiquitination and polyubiquitination. When a single ubiquitin molecule is added to a substrate's lysine residue, monoubiquitination occurs. In polyubiquitination, ubiquitin molecules are added to a single ubiquitin molecule to form polyubiquitin chains.^14^^,^^15^ Polyubiquitin chains K48 and K11 mainly mediate proteasomal degradation. However, K63-linked polyubiquitination, which is typically less common in tumors, is usually not involved in proteasomal degradation but is associated with cellular signal assembly and transduction and repair of damaged cells.^16^^,^^17^ The substrate protein is labeled with a ubiquitin molecule and then degraded in the 26s proteasome. The ubiquitin-proteasome system (UPS) includes three classes of ubiquitinates E1 ubiquitin activating enzymes (E1s), E2 ubiquitin binding enzymes (E2s), and E3 ubiquitin ligases (E3s). Based on the supply of ATP, E1 transfers activated ubiquitin molecules to E2. Bound ubiquitin molecules interact with E2 to transfer the ubiquitin molecules to E3.^18^ In this process, E3 ubiquitin ligases play a substantial role. E3 ubiquitin ligases can be categorized into three families based on their structural characteristics and operational mechanism: RING (really interesting new gene) E3s, HECT (homologous to E6-AP carboxyl terminus) E3s, and RBR (RING-between-RING) E3s.^19^ RING E3s transfer ubiquitin molecules from E2 to the lysine of the substrate, while HECT and RBR E3 ligases can catalyze the transfer of ubiquitin molecules from E2 to the cysteine of the substrate.^20^ In deubiquitination, ubiquitin molecules are removed from the substrate using deubiquitinating enzymes. The deubiquitinase protein family removes ubiquitin molecules from substrate proteins by hydrolyzing the peptide or isopeptide bonds at the carboxyl-terminal end of the ubiquitin, which is opposite to the functions of E3 ubiquitin ligase.^21^ Based on their sequence and structural domain characteristics, these deubiquitinating enzyme classes can be categorized into five families: UCH (ubiquitin carboxy-terminal hydrolases) family, USP/UBP (ubiquitin-specific protease and ubiquitin-binding protein) family, OTU (ovarian tumor proteases) family, MJD (Machado-Joseph domain) family, and JAMM (JAB1/MPN/MOV34) family.^22^ Ubiquitination modification, as a critical post-translational protein modification (Fig. 1), can regulate tumor progression by targeting various TME-related cells and proteins.

## Ubiquitination modification regulates tumor cells in the TME

### Ubiquitin modification mediates cell cycle progression

**Figure 2:**
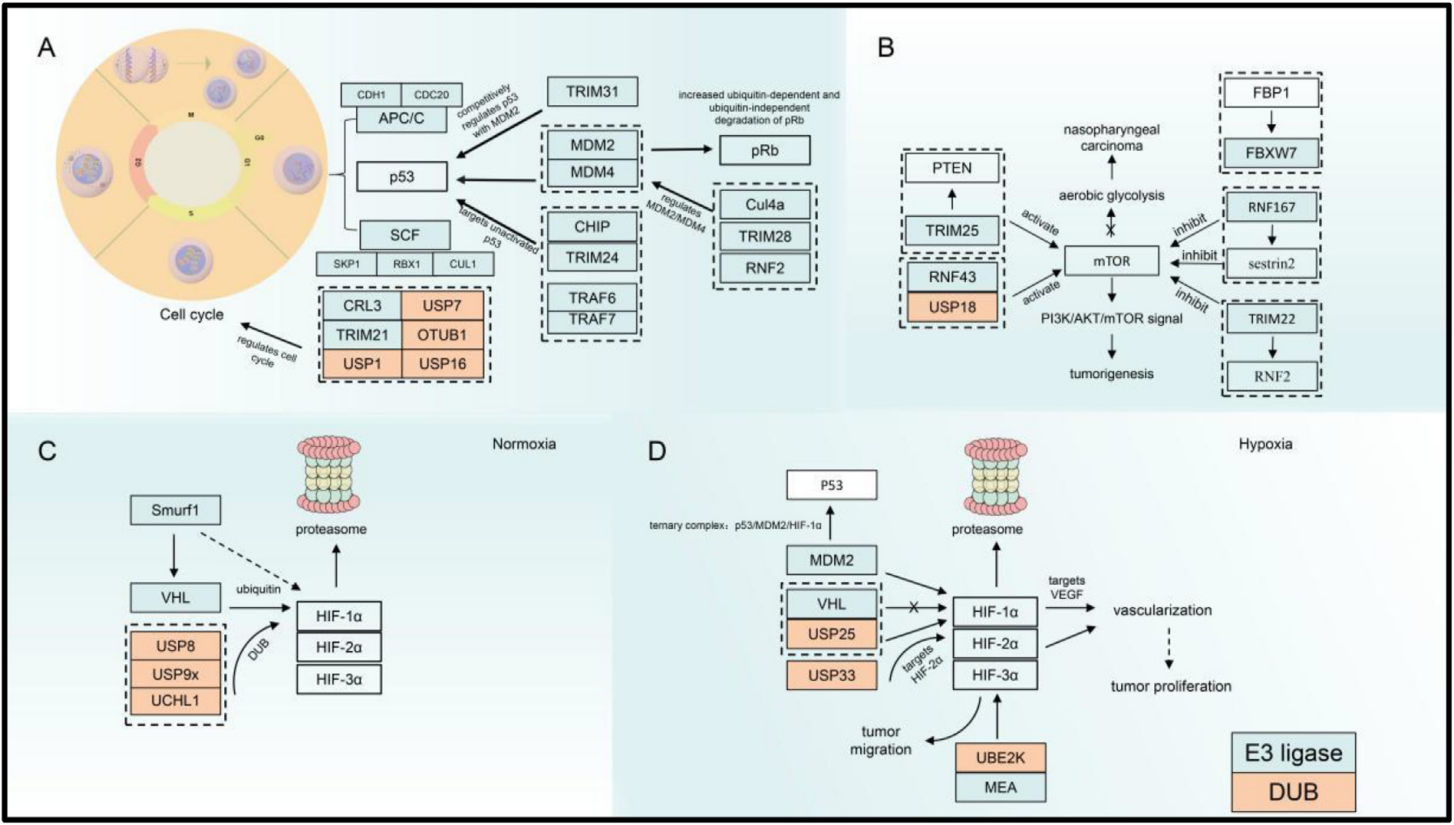
UPS regulates tumor cells. **(A)** E3 ubiquitin ligases APC/C and SCF regulate the cell cycle. MDM2, as a classical ubiquitin ligase, regulates p53 to regulate the cell cycle, while TRIM2, another E3 ligase, competes with it. Other ubiquitin ligases such as Cul4a, TRIM28, and RNF2 also regulate MDM2 ubiquitin ligase, which indirectly regulates p53 protein stability. In addition, several ubiquitin ligases including CHIP, TRIM24, TRAF6, and TRAF7 can also regulate p53. **(B)** TRIM25, FBP1, RNF167, and TRIM22 regulate the mTOR signaling pathway by modulating PTEN, FBXW7, sestrin2, and RNF2, respectively, which ultimately regulates energy metabolism in tumor cells. **(C, D)** HIF-1α is regulated by different ubiquitin proteases under hypoxia and normoxia conditions respectively. APC/C, anaphase-promoting complex/cyclosome; CDC20, cell division cycle 20; SCF, Skp1-Cul1-F-box; CDH1, E-Cadherin; SKP, S-phase kinase-associated protein; TRIM, tripartite motif-containing; MDM, mouse double minute; RBX/RNF, RING finger protein; CRL, cullin-RING E3 ubiquitin ligase; USP, ubiquitin-specific protease; OTU, ovarian tumor proteases; mTOR, mechanistic target of rapamycin complex; FBXW, F-Box, and WD repeat domain containing; FBP1, fructose-bisphosphatase 1; UCH, ubiquitin carboxy-terminal hydrolases; VHL, Von Hippel-Lindau protein; HIF, hypoxia-inducible transcription factor; UBE2K, ubiquitin-conjugating enzyme E2K; MAEA, macrophage-erythroblast attacher; VEGF, vascular endothelial growth factor; DUB, deubiquitinase; TRAF, tumor necrosis factor receptor-associated factor.

Tumor cells undergo uncontrolled rapid proliferation and metastasis, which is a characteristic that distinguishes them from normal cells. The entry of cells into the cell cycle for mitosis in order to generate new cells requires the involvement of various cyclins/cyclin-dependent kinases to ensure normal cell proliferation. Ubiquitination, a post-transitional modification, is crucial for regulating the stability of various cyclins and cyclin-dependent kinases during cell cycle progression. Two important E3 ligases such anaphase-promoting complex/cyclosome (APC/C) and Skp1-Cul1-F-box (SCF) play crucial roles in regulating cell cycle proteins^23^^,^^24^ (Fig. 2A). These two ubiquitin ligases are Cullin RING E3 ligase family members. APC/C is linked to the complex consisting of a scaffold Cullin-like protein APC2 and a coactivator subunit. This ubiquitin ligase regulates G1 phase cell activity by binding to coactivators cell division cycle 20 homolog and e-cadherin (CDH1) and subsequently regulates mitotic progression.^24^ SCF contains an adaptor protein SKP1, scaffold protein CUL1, and a RING finger protein 1 (RBX1/RNF1) recruiting E2, which together address DNA damage in the cell cycle by binding to other ubiquitin ligases including FBXW7 (WD repeat domain containing 7), β-Trcp (β-transducin repeat-containing proteins), and SKP2.^25^ Two ubiquitin ligases also interact. For example, the SCF/SKP 2 axis regulates APC/CDH1-mediated C-terminal binding protein interacting protein degradation to regulate p-RB in the G2 phase by inhibiting transcriptional gene responses of the E2F complex and regulating the stability of the cyclin/cyclin-dependent kinase inhibitor p27 by cooperating with SCF/SKP2 axis and APC/CDH1 to induce G2 retardance.^26^ Thus, the imbalance in the expression of APC/C and SCF and SCF-associated ubiquitin ligases may affect the cell cycle, which thus affects cell proliferation and mediation of tumorigenesis.^27^^,^^28^ For example, USP10 can up-regulate tumor development in esophageal squamous cell carcinoma by modifying cyclin Anillin in concert with the CDH1 of APC/C.^29^ p53 is a key protein detected in the G1 phase and even in the whole cell cycle. It is a well-known tumor suppressor gene.^30^^,^^31^ The majority of tumorigenesis is associated with p53 mutations.^32^ Mouse double minute 2 (MDM2) functions as a classical ubiquitin ligase that regulates p53 protein degradation. MDM2 can undergo self-ubiquitination, but such ubiquitination is unstable and may cause aberrant p53 activation.^33^ MDM4 (also known as MDMX) interacts with the MDM2 protein to ensure that p53 transcriptional activity is normal.^34^ Furthermore, ubiquitin ligases such as tripartite motif-containing 28 (TRIM28), RNF2, and Cul4a can also promote p53 degradation by cooperating with MDM2.^35^, ^36^, ^37^, ^38^ Other ubiquitin-related enzymes such as TRIM31 can form a competitive relationship with MDM2 and prevent MDM2 from interacting with p53, leading to p53 activation in breast cancer.^39^ E3 ligases such as TRIM24, TNF receptor-associated factor 6 (TRAF6), TRAF7, and C terminus of Hsc70-interacting protein (CHIP) maintain a low cellular p53 expression in the absence of signal activation of p53 genes^40^, ^41^, ^42^, ^43^ (Table 1). Because of the special properties of the MDM2/p53 axis, most p53-related inhibitors, such as PROTAC, were developed based on this axis.^44^ Other ubiquitin ligases are associated with the cell cycle such as TRIM21, USP1, and USP7 in cancer development.^45^, ^46^, ^47^

**Table 1:** Summary of ubiquitination enzyme regulation of targeting proteins.

Enzyme	Name	Targets	Cancer	Animal models	Reference
E3 ligase	APC/C	G1 period	/	/	23
	SCF	DNA damage	/	/	24
E3 ligase	FBXW7	SCF	/	/	25
	β-Trcp		/	/	
	SKP2		/	/	
DUB	USP10	ANLN	Esophageal squamous cell carcinoma	Human	29
E3 ligase	MDM2	P53	Neuroblastoma	Mice	33
	MDM4		Neuroblastoma	Mice	34
	TRIM28		Melanoma	/	36
	TRIM31		Breast cancer	Mice	39
	RNF2		Ovarian tumor	Mice	37
	Cul4a		Breast cancer/liver cancer	Mice	38
	TIRM24		Breast cancer	Drosophila	40
	TRAF6		Lung cancer	Mice	41
	TRAF7		Breast cancer	/	42
	CHIP		Lung cancer	Mice	43
E3 ligase	TRIM25	PTEN	Non-small cell lung cancer	Mice	50
E3 ligase	RNF43	p85	Colorectal cancer	Human	51
DUB	USP18	mTOR	Ovarian cancer	Human	52
E3 ligase	RNF167	Sestrin2	Colorectal cancer	Human	53
E3 ligase	TRIM22	NRF2	Osteosarcoma	Human	54
E3 ligase	FBXW7	mTOR	Nasopharyngeal carcinoma	Human	25
E3 ligase	Smurf1	VHL	Many types of cancer	/	60
DUB	VHL	(HIF-1α) normoxia	Renal cancer	Mice	62
	USP8		Non-small-cell lung cancer	Mice	61
	USP9x		Pancreatic cancer, gastric cancer	Mice	60
	UCH-L1		Ovarian cancer	Mice	63
E3 ligase	MDM2	(HIF-1α) Hypoxia	Mesothelioma, ovarian cancer	Mice	67

### Ubiquitination regulates energy metabolism in cells

Based on the particularity of the TME, energy metabolism, including glucose metabolism, is mostly enhanced in tumor cells.^48^ Mechanistic targets of rapamycin complex (mTOR) which consists of mTOR1 and mTOR2 mutation are involved in most cancer.^49^ For example, TRIM25 down-regulates the activity of this protease through polyubiquitination modification of PTEN in non-small cell lung cancer, thus activating the PI3K/mTOR pathway to promote tumor development (Fig. 2B). Other ubiquitination modifications can also exert a tumor growth-promoting role by enhancing the mTOR signaling pathway.^50^ Mutations in RNF43 G659fs are frequently found in colorectal cancer, and RNF43 G659fs mutations can bind to p85 and thus enhance p85 ubiquitination, leading to mTOR signaling activating. How p85 ubiquitination is regulated remains unclear.^51^ The deubiquitination enzyme USP18 can also up-regulate ovarian cancer development in ovarian cancer by activating the AKT/mTOR signaling pathway through direct regulation of mTOR and AKT proteins.^52^ In a few cancers, mTOR is also inhibited through ubiquitin modification. The E3 ligase RNF167 cooperates with STAM-binding-protein-like 1 to modify an amino acid sensor, sestrin2. This sensor transacts amino acid signals to mTOR1 and in turn, activates the mTOR signaling pathway. When sestrin2 ubiquitination increases, it inhibits mTOR signaling in colon cancer.^53^ Other ubiquitin ligases, such as TRIM22, can accelerate nuclear factor erythroid 2-related factor 2 (NRF2) degradation and thus regulate mTOR signaling. In osteosarcoma, down-regulated TRIM22 expression led to increased stability of the NRF2 protein and inhibition of the mTOR-associated autophagy signaling pathway, thereby triggering cancer development.^54^ Based on the important role of the mTOR signaling pathway, developing mTOR-related inhibitors seems extremely crucial. In nasopharyngeal carcinoma, fructose-1, 6-bisphosphatase 1 inhibits autologous ubiquitination of the E3 ligase FBXW7, thereby stabilizing this ubiquitin ligase, promoting FBXW7 to regulate mTOR protein ubiquitination, inhibiting the mTOR signaling pathway to inhibit glycolysis, and promoting radiation-induced apoptosis and DNA damage for tumor growth inhibition^55^ (Table 1).

### Ubiquitination regulates angiogenesis

Because tumor cells require a large amount of nutrition from the TME, stromal cells in the TME would be nutrient-deficient, and the function of related stromal cells would be inhibited, thereby leading to tumor cell proliferation.^56^ Rapidly proliferating tumor cells stimulate angiogenesis, but the uneven distribution of the new tumor vasculature results in the uneven distribution of oxygen, which makes the TME present a temporary or permanent hypoxic state.^57^ Being a regulator, it can guide rapid tumor cell vascularization, offering oxygen and nutritional conditions for tumor cell growth and metastasis and enabling cancer cells to rapidly adapt to severe hypoxic conditions.^56^ Along with hypoxia-inducible factor 1α (HIF-1α), HIF-2α, and HIF-3α are members of the HIF family. HIF-1α is the most sensitive to the oxygen content in the TME.^57^ The HIF-1α content is low under normal oxygen conditions (Fig. 2C), which because this protein can be targeted through ubiquitination by E3 ubiquitin ligases such as Von Hippel Lindau (VHL) to mediate its degradation. This is a complex process involving other post-translational modifications. Hydroxylation-mediated changes in the two aerobic-dependent hydroxyprolines of HIF-1α indicate that HIF-1α can be modified through ubiquitination so that it enters the proteasomal degradation pathway.^58^ Other E3 ligases such as Smad ubiquitylation regulatory factor 1 would also participate in regulating VHL stability under normal conditions.^59^ Some deubiquitinases such as USP8, USP9X, and UCHL1 can participate in VHL-mediated ubiquitination modification of HIF-1α.^60^, ^61^, ^62^ However, under hypoxia, VHL ubiquitination can no longer modify HIF-α (Fig. 2D), which leads to HIF1-α stabilization, thereby mediating vascularization activity and rapid tumor growth.^63^ USP25 can regulate HIF-1α-associated transcription factors under severe hypoxia, regulating cancer development.^64^ MDM2 can participate in the regulation of p53 protein stability. It can also directly ubiquitinate HIF-1α. According to some reports, MDM2, p53, and HIF-1α form a ternary complex, leading to MDM2 degradation in a p53-dependent manner^65^, ^66^, ^67^ (Table 1). Several ubiquitin-related enzymes also regulate HIF-2α protein stabilization; for example, in gliomas, USP33 modifies HIF-2α through deubiquitination to promote angiogenesis and cancer progression.^68^ HIF may also regulate other activities of cancer cells in tumor development. For example, the ubiquitin-conjugating enzyme E2K could increase HIF expression in hepatocellular carcinoma, promoting tumor cell proliferation and migration.^69^ HIF-1α can also be up-regulated by the E3 ligase macrophage-erythroblast attacher, leading to the proliferation of tumor cells and elevated migration capacity in glioblastoma.^70^

## Ubiquitination modification regulates immune cells in the TME

### Ubiquitination modification mediates the T cell function in the TME

CD4^+^ and CD8^+^ T cells can be differentiated into the corresponding T-helper (Th1, Th2, Th9, Th17) and regulatory T cells and cytotoxic T-lymphocytes respectively, under the stimulation of corresponding major histocompatibility complex (MHC) molecules.^71^^,^^72^ T-helper, by recognizing MHC-II antigens on dendritic cells, secretes inflammation-related factors such as interleukin2 (IL-2) and interferon (IFN-γ).^73^ Regulatory T cells, which can be differentiated from CD4^+^, secrete IL-2 which regulates the homeostasis and function of natural killer cells.^74^

### Ubiquitination regulates PD-1/PD-L1

**Figure 3:**
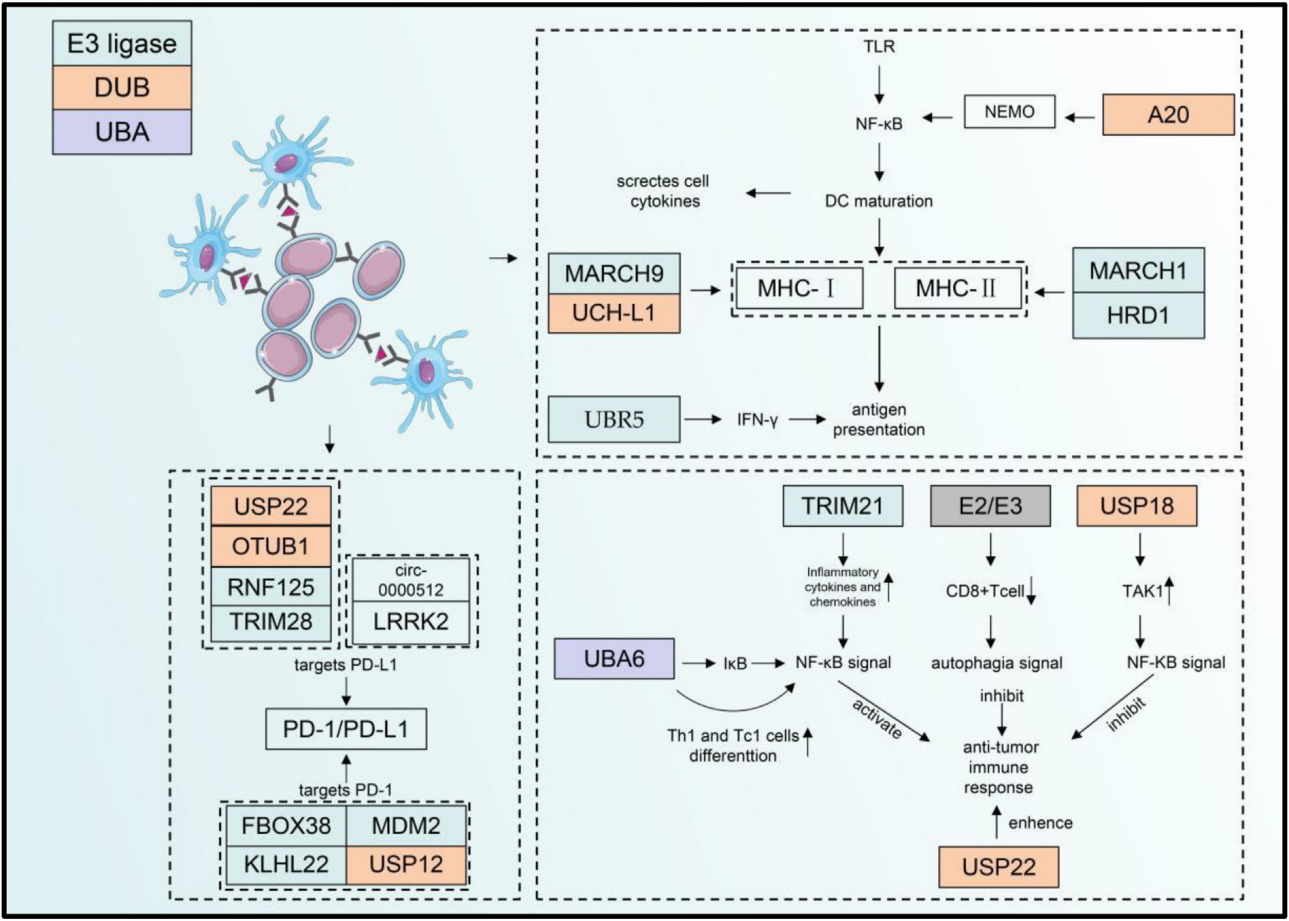
UPS regulates immune cells in the TME. DCs contact T cells via MHC to transmit antigen information. The dashed box proximal to DCs depicts the mechanism by which ubiquitination modulates DCs. Ubiquitin enzymes MARCH 1, UCH-L1, MARCH9, and HRD1 regulate MHC-I and MHC-Ⅱ respectively. The results of MARCH1 regulating MHC-Ⅱ may affect the stabilization of MHC-Ⅰ. Ubiquitin-editing enzyme A20, which exerts a deubiquitinating function, mediates the maturation of DCs by regulating NEMO in the NF-κB signaling pathway of DCs. UBR5 also mediated the antigen presentation function of DCs through the regulation of IFN-γ protein stability. PD-1/PD-L1 could be regulated by DUBs and E3 ubiquitin ligases such as USP22, OTUB1, and USP12. USP can regulate T cells by autophagy and NF-κB signaling which ultimately regulate their anti-tumor immune response of them. DCs, dendritic cells; TLR, Toll-like receptor; NF-κB, nuclear factor kappa B; NEMO, nuclear factor-kappa B essential modulator; MARCH, membrane-associated ring–CH–type finger 1; HRD, 3-hydroxy-3-methylglutaryl reductase degradation1; UBR, the ubiquitin-binding region; UCH, ubiquitin carboxy-terminal hydrolases; MHC-Ⅰ/Ⅱ, major histocompatibility complex Ⅰ/Ⅱ; IFN-γ, interferon-γ; USP, ubiquitin-specific protease; OTU, ovarian tumor proteases; RNF, RING finger protein 1; TRIM, tripartite motif-containing; LRRK, eucine-rich repeat kinase; FBOX, F-box-containing protein 38; KLHL, Kelch-like family member; MDM, mouse double minute; UBA, ubiquitin-like modifier activating enzyme.

PD-1, an inhibitory receptor of T cells, acts as a crucial checkpoint for immune escape. PD-1 and its ligands PD-L1 or PD-L2 play an extremely significant role in regulating tumor progression.^75^ Aberrant ubiquitination and deubiquitination of this checkpoint affect checkpoint-mediated immune activity.^76^ F-box only proteins 38 (FBXO38) is a PD-1-specific E3 ligase and mediates polyubiquitination of the K233 site on PD-1, thereby reducing PD-1 expression on the T cell surface and blocking PD-1/PD-L1 axis-mediated immunosuppression (Fig. 3). In FBXO38 conditional knockout mice, PD-1 levels were elevated in tumor-infiltrating T cells, which resulted in more rapid tumor development in the mice.^77^ Kelch like family member 22 (KLHL22), another E3 ligase of the BTB-CUL3-RBX1 complex, can specifically recognize the substrate and mediate ubiquitination. This ligase mediates PD-1 degradation before translocating to the T cell surface. A marked decrease in the level of this ubiquitin ligase in the tumor-infiltrating T cells led to PD-1 overaccumulation and T cell suppression.^78^ USP12 also regulates PD-1 stabilization in cancer development.^79^ MDM2, an E3 ubiquitin ligase of PD-1, can promote PD-1 degradation through ubiquitination of disaccharidased PD-1 and enhance the anti-tumor effect of T cells.^80^ Along with the regulation of PD-1 in T cells, the ubiquitination system targets PD-1 in tumor-associated macrophages, thereby regulating overall tumor growth and development. In macrophages, the E3 ubiquitin ligase c-Cbl induces ubiquitination degradation by interacting with the PD-1 tail, thus ultimately improving the phagocytic ability of macrophages and exerting anti-tumor effects.^81^ Tumor cells also regulate the ubiquitination of PD-L1, a PD-1 ligand, altering the expression of leucine-rich repeat kinase 2, ring finger protein 125, TRIM28, circ-0000512, USP22, OTUB1, *etc*.^82^, ^83^, ^84^, ^85^, ^86^, ^87^ (Table 2). In summary, ubiquitination is crucial for regulating the PD-1/PD-L1 axis. Ubiquitination allows it to be combined with immune checkpoint inhibitors, such as PD-1/PD-L1, thereby improving patient response rates and treatment effects. Of note, although multiple studies have reported the regulatory effect of ubiquitination on the PD-1/PD-L1 protein level, it is not an isolated event but is closely related to other post-translational modifications. For example, MDM2 mainly promotes the ubiquitination of deglycosylated PD-1 to down-regulate its protein levels.^80^ This suggests that studies should focus more on the overall concept of cells during research. In the future, researchers may concentrate more on the synergistic effects of ubiquitination and other post-translational modifications of proteins in order to improve the intervention efficiency.

**Table 2:** Summary of ubiquitination regulation of targeting proteins.

Enzyme	Name	Targets	Cancer	Animal models	Reference
E3 ligase	FBXO38	PD-1	B16F10 melanoma	Mice	78
	KLHL22	PD-1	Cub cutaneous melanoma	Mice	79
	MDM2	PD-1	Colorectal cancer	Mice	81
	c-Cbl	PD-1 (macrophages)	Colorectal cancer	Mice	82
DUB	USP12	PD-1	Lung cancer	Mice	80
E3 ligase	RNF125	PD-1	Head and neck squamous cell carcinoma	Mice	84
	TRIM28	PD-1/TBK1	Gastric cancer	Mice	86
DUB	USP22	PD-L1	Pancreatic cancer	Mice	88
	OTUB1		Murine breast cancer	Mice	87
DUB	USP18	TAK1	/	/	89
	USP22	T cell	Pancreatic cancer	Mice	92
E1 activating enzyme	UBA6	IκBα (T cells)	Lupus	Mice	93
E3 ligase	UBR5	IFN-γ	Triple-negative breast cancer	Mice	104
	MARCH1	MHC-II	/	Mice	97
	MARCH9	MHC-I	/	Mice	98
	HRD1	BLIMP-1	/	Mice	99
DUB	A20	NEMO	Dendritic cells	Mice	101
	UCH-L1	MHC-I	Listeria	Mice	100
	OTUD6A	NLR3			114
E3 ligase	Praja2	MFHAS1	Malignant fibrous histiocytoma	Mice	109
	Pellino-1	K63 of IRAK1	Melanoma	Mice	110
	FBXW7	c-Myc	Lewis lung carcinoma cells	Mice	112
	ITCH	Macrophages	/	Mice	111
	TRIM24	Macrophages	Breast cancer	Mice	113
	CRL4	CD47	Multiple myeloma	Mice	120
	UBR	SHP-2	Many types of cancer	/	121
DUB	Mysm1	Macrophages	/	Mice	115
	OTUD5	YAP	Triple-negative breast cancer	Mice	116
DUB	USP12	p65	Colorectal cancer	Mice	126
E3 ligase	TRAF6	STAT3 of k63	Lung cancer	Mice	127

### Ubiquitination regulates other T-cell functions

Ubiquitination modification also regulates other T-cell functions. In the presence of androgens, the protein level of USP18, a deubiquitination enzyme, in T cells is up-regulated. This enzyme promotes transforming growth factor-beta (TGF-beta)-activated kinase 1 deubiquitination and inhibits TAK1 phosphorylation, and subsequent activation of the NF-κB signaling pathway, which ultimately induces the inhibition of the anti-tumor effect of T cells.^88^ In addition, in a study of oral lichen planus, TRIM2, a ubiquitination ligase, also ubiquitinated NF-κB and activated its signaling pathway, ultimately up-regulating the inflammatory function of T cells. This suggested that targeting TRIM2 helps regulate the anti-tumor effect of T cells.^89^ Moreover, E2–E3 ubiquitin ligases in T cells were disrupted in patients with renal metastatic cancer, which led to autophagy defects in circulating and tissue-resident CD8^+^ memory T cells and ultimately resulted in dysfunction and apoptosis.^90^ In addition to directly affecting T cells, ubiquitination can indirectly affect T cell function through the regulation of ubiquitination in tumor cells. The decreased expression of the deubiquitinase USP22 in pancreatic cancer cells promoted the infiltration of natural killer and T cells, thereby enhancing the anti-tumor immune response of the TME.^91^ Ubiquitination modification also regulates T cells to promote the differentiation of other cells. E1 ubiquitin-activating enzyme UBA6 increases p65 activation in the NF-κB signaling pathway of T cells by accelerating IκBα degradation. UBA6 regulates IFN-γ stability by modulating p65 of the NF-κB signaling pathway to promote Th1 and Tc1 cell differentiation^92^ (Table 2). Ubiquitination has a crucial regulatory role in the validation function and anti-tumor effect of T cells. It has a crucial impact on the survival of memory CD8^+^ T cells. However, the underlying mechanism remains unclear. If the regulatory action of ubiquitination-related enzymes on memory CD8^+^ T cells can be clearly studied, the findings may have a great effect on improving anti-tumor immunity.

### Ubiquitination mediates DC maturation and function in the TME

DCs are crucial for the immune system. They play a vital role in connecting innate and adaptive immunity. These cells can drive adaptive immunity through antigen presentation and regulate the activity of innate immune cells by secreting immunostimulatory cytokines.^93^ MHC-I molecules load and present endogenous peptides to CD8^+^ T cells through different intracellular pathways. This is of great significance for the anti-tumor function of T cells. By contrast, MHC-II molecules load and present most exogenous peptides to CD4^+^ T cells. Furthermore, endogenous peptides in DCs can also be presented to CD8^+^ T cells using a cross-presentation approach.^94^ Therefore, the antigen presentation function of DCs is of great significance for the anti-tumor immune response of immune cells in the TME. MARCH1 can mediate the ubiquitination of MHC-II molecules on the DC surface (Fig. 3). This ubiquitin ligase regulates MHC II stability through ubiquitination at the tail of the MHC-II β chain. Then, the expression of MHC-II molecules and CD86 on the DC surface was regulated, thereby suppressing T-cell activation.^95^^,^^96^ Moreover, MARCH1-mediated regulation of MHC-II affected the maturation of MHC-I stabilization. A specific relationship exists between MHC-I and MHC-II. MARCH1-mediated MHC-II ubiquitination affects the antigen presentation pathway of MHC-I. MHC-I expression was reduced in MARCH1-deficient DCs. MARCH1 does not directly regulate MHC-I. It is indirectly induced through MHC-II ubiquitination.^96^ MARCH9, another ubiquitin ligase, regulates MHC-I ubiquitination. This transmembrane protein depends on lysine residues in the cytoplasmic tail for its ubiquitination function. MARCH9 plays a key role in regulating the entry of MHC-I into nucleosomes and MHC-I-mediated antigen presentation.^97^ The E3 ligase 3-hydroxy-3-methylglutaryl reductase degradation 1 (HRD1) regulates ubiquitination modification of B lymphocyte-induced maturation protein 1, a transcription factor for MHC-II in DCs, thereby promoting MHC-II transcription and affecting CD4^+^ T cell activation in the inflammatory response.^98^ UCH-L1 can regulate antigen cross-presentation pathway by promoting the recycling of MHC-I molecules in DCs. MHC-I at the cytoplasmic membrane or endoplasmic reticulum is recruited during antigen cross-presentation for phagosomal-cytoplasmic and vesicular cross-presentation pathways. Subsequently, some peptides derived from external pathogen molecules are loaded onto MHC-I in phagosomes and then shuttled to the plasma membrane for presentation and act as MHC-I/AG complexes. UCH-L1 deficient DCs present with reduced MHC recycling capacity. UCH-L1 deficient mice have a significantly reduced ability of antigen cross-presentation to cytotoxic T-lymphocytes *in vivo* and *in vitro* after infection with Listeria monocytogenes.^99^ A20, a deubiquitinase targeting NEMO of DCs, up-regulates the maturation and cytokine production of DCs. A20 deficiency can lead to the development of autoimmune defects.^100^, ^101^, ^102^ Ubiquitin ligase UBR5 does not directly target DCs in triple-negative breast cancer. However, IFN-γ expression increased in UBR5 knockout 4T1 tumor-bearing mice could enhance the antigen-presenting ability of DCs, promoting treatment and presentation of DCs to T cells, and triggering a specific immune response to a tumor to inhibit tumor growth^103^ (Table 2). In summary, ubiquitination significantly affects MHCI/II protein levels in DCs, which can subsequently affect the activation and anti-tumor function of T cells by impacting the antigen-presenting ability of DCs. Current research in this area is focused on exploring mechanisms, and gaps remain in how to intervene. Follow-up research is warranted to determine how to enhance the antigen-presenting ability of DCs and activate T cells by interfering with DC ubiquitination.

### Ubiquitination mediates tumor-associated macrophage polarization and phagocytosis in the TME

**Figure 4:**
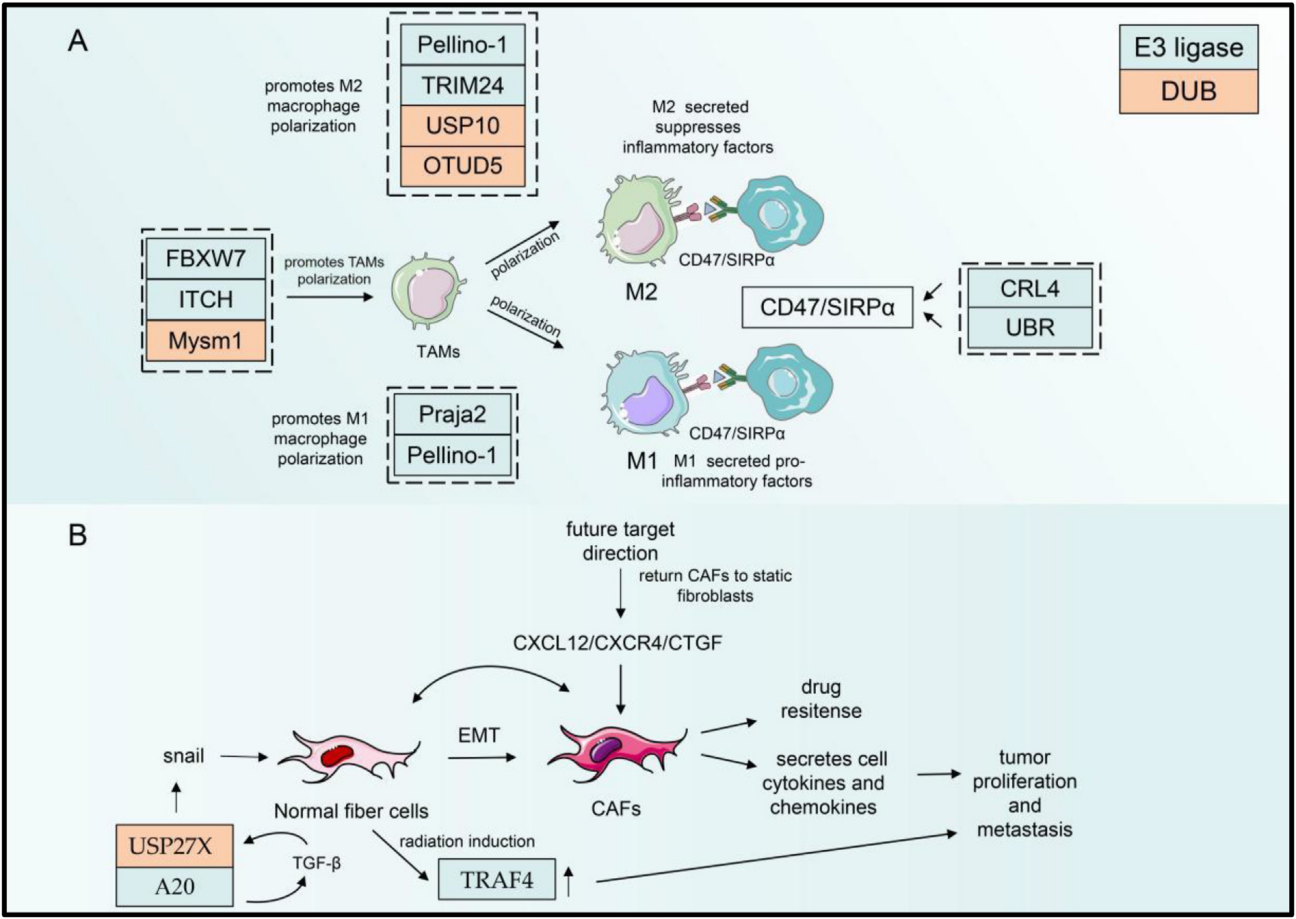
UPS regulates TAMs and CAFs in the TME. (A) Ubiquitination modification regulating macrophage polarization. Macrophages receiving different signals can be polarized into macrophages of M1 and M2. Ubiquitin enzymes that regulate macrophage polarization are shown in the figure. The polarization of tumor-associated macrophages can be regulated by UPS such as FBXW7, ITCH, and Mysm1. The ubiquitination enzymes CRL4 and UBR can regulate CD47/SIRPα to mediate tumor immune response in TAMs. (B) The transformation of normal fibrocytes to tumor-associated fibroblasts through regulation by snails can be regulated by UPS such as USP27X. CAFs could also transform into normal fibrocytes by regulation of CXCL12/CXCR4/CTGF. FBXW, F-Box and WD repeat domain containing; USP, ubiquitin-specific protease; TRIM, tripartite motif-containing; OTU, ovarian tumor proteases; CRL, cullin-RING E3 ubiquitin ligase; UBR, the ubiquitin-binding region; SIRP, CD47-signal-regulatory protein; EMT, endothelial-mesenchymal transition; TRAF, tumor necrosis factor receptor-associated factor; CAFs, cancer-associated fibroblasts; CXCL, C-X-C motif chemokine ligand; CTGF, connective tissue growth factor.

Macrophages are among the most crucial cells in the tumor immune microenvironment. They can be roughly categorized into two polarization directions, M1 (anti-tumor macrophages) and M2 (pro-tumor macrophages).^104^, ^105^, ^106^ However, in reality, the functions of macrophages are far from simple, and the macrophage population has strong heterogeneity and plasticity. In tumors, macrophages often tend to be M2-like macrophages. Therefore, targeting the elimination of M2-like macrophages or transforming them into M1-like macrophages is the main research direction in cancer treatment. Moreover, macrophages have a specialized and significant antigen-presenting function. In lung adenocarcinoma, under the action of microRNAs secreted by tumor cells, macrophages exhibit inhibition of the ubiquitination and degradation of misshapen-like kinase 1 through a series of pathway reactions (Fig. 4A), which ultimately activates the downstream c-Jun N-terminal kinase signaling pathway and polarizes the macrophages toward M2-like macrophages and thus promotes tumor progression.^107^ The E3 ubiquitin ligase Praja2 catalyzes ubiquitination of the modified malignant fibrous histiocytoma amplified sequence 1 (MFHAS1).^108^ This protein can activate JNK/p38 and NF-κB pathways to promote M1 macrophage polarization and inflammatory responses.^108^ Pellino-1, an E3 ubiquitin ligase, regulates M1 macrophage polarization. However, new studies have demonstrated that Pellino-1 can inhibit IL-10-mediated M2 macrophage polarization by regulating k63 ubiquitination of IL-1 receptor-associated kinase 1 to activate signal transducer and activator of transcription 1 (STAT1) in response to IL-10 stimulation.^109^ Some other E3 ligases such as FBXW7, itchy E3 ligase, and TRIM24 can also regulate macrophage polarization^110^, ^111^, ^112^ (Table 2).

OTUD6A, a deubiquitination enzyme, in macrophages, can up-regulate NLRP3 protein levels through deubiquitination, which elevates IL-1β levels, ultimately enhancing the inflammatory function of macrophages.^113^ Another deubiquitinating enzyme Myb-like, SWIRM, and MPN domains 1 (Mysm1) regulates macrophage survival and polarization. Mysm1-deficient macrophages produce more pro-inflammatory factors including IL-1β, TNFα, and iNOS, and sustained phosphorylation of AKT, a major PI3K target, can be detected. However, the exact mechanism of how Mysm1 regulates macrophage polarization remains unknown.^114^ The deubiquitinase enzyme OTUD5 mediates YAP deubiquitination, thereby stabilizing the protein to promote M2 macrophage polarization. M2 macrophages with high YAP expression enhance the cellular invasive capacity of cancer cells, thereby improving the progression of triple-negative breast cancer^115^ (Table 2).

Phagocytosis and antigen presentation are vital functions for macrophages to exert their anti-tumor effects. However, during interactions with macrophages, tumor cells often transmit the “don't eat me” signal to evade macrophage phagocytosis. For example, tumor cells can express CD47 to interact with SIRPα on the macrophage surface and mediate immune escape.^116^, ^117^, ^118^ CD47 can be ubiquitinated by DDB1-CUL4A, which then blocks the CD47/SIRPα immune checkpoint and improves the anti-tumor immune response.^119^ UBR also regulates the CD47/SIRPα axis during immune therapy^120^ (Table 2). In summary, ubiquitination is crucial for regulating macrophage polarization and function. This regulatory effect occurs in already existing tumors as well as in some precancerous lesions of tumors.^113^ Thus, macrophage ubiquitination can not only serve as a target for anti-tumor therapy but also prevent tumor occurrence.

### Ubiquitination mediates MDSC function in the TME

MDSCs are an immature population of immune cells, which differentiate into DCs, macrophages, and neutrophils.^121^ MDSCs secrete high NO, Arg1, iNOS, and ROS concentrations, which inhibit immune cells in the TME, especially T cells, promote tumor cell growth, and cause tumor immune escape.^122^ Targeting MDSCs is likely to be a breakthrough therapy against tumors in the future.^123^ MDSCs can be simply divided into two subgroups based on their surface marker: granular or polytype nucleoid (PMN-) and mononuclear (M−) MDSCs. The series of chemokines secreted by M-MDSCs can promote regulatory T-cell proliferation and differentiation to inhibit the immune microenvironment.^124^ USP12 can regulate p65 deubiquitination in the NF-κB signaling pathway in MDSCs, thereby mediating PD-L1 and iNOS expression and the anti-tumor immune response of CD4^+^ T cells. At the same time, USP12 can affect INF-γ stability and reduce the anti-tumor immune capacity in the TME.^125^ TRAF6, another member of the ubiquitin ligase family, modifies K63 polyubiquitination and STAT3 phosphorylation, thereby affecting MDSC differentiation. Examples of MDSC ubiquitination are few. More ubiquitination regulatory proteins will be identified in future studies.^126^

## Ubiquitination mediates the function of non-immune cells in the TME

### Ubiquitination mediates the function of tumor-associated fibroblasts in the TME

Tumor-associated fibroblasts (CAFs) are the most abundant in stromal cells in tumors. They secrete cytokines and chemokines to enhance the proliferation and metastasis of malignant tumors.^127^ CAFs have a wide range of sources. During the transition from normal fibroblasts to CAFs, snail plays a crucial role as a transcription factor regulating cell protein expression and cytokine secretion.^128^ TRAF4, which is highly expressed in normal lung fibroblasts after radiotherapy (Fig. 4B), interacts with NADPH oxidase-2 (NOX2) and NOX4, thereby delaying lysosomal-dependent degradation. NOX2 and NOX4 localization in endosomes is stabilized and can activate the NF-κB signaling pathway in healthy cells of the lung, increasing ICAM1 secretion and non-small cell lung cancer invasion.^129^ In invasive basal-like breast cancer cells, the ubiquitin editing enzyme A20 promotes tumor migration by modifying the monoubiquitination of three lysines in snails to promote transforming growth factor-β (TGF-β)-induced epithelial-mesenchymal transition in invasive basal-like breast cancer cells.^130^ USP27X expression was positively correlated with snails. TGF-β-activated USP27X can serve as a deubiquitinating enzyme and stabilize snails, and the decreased USP27X expression leads to the inhibition of TGF-β-induced activation of epithelial-mesenchymal transition and fibroblasts.^128^ Also, reports have proposed that activated CAFs are recovered to normal static fibroblasts by targeting signaling pathway downstream molecules, such as C-X-C motif chemokine ligand 12 (CXCL12), CXCR4, and anti-connective tissue growth factor (CTGF).^131^, ^132^, ^133^ Therefore, these results all implied that targeting CAFs can be a future direction for tumor treatment (Table 3). Targeting CAFs is of great significance in regulating the TME, especially that related to tumor invasion and metastasis. The expression of its related proteins may serve as both a target for subsequent research about anti-tumor therapy and an important indicator for judging tumor prognosis. The expression of CAF-related proteins may serve as both a target for subsequent research about anti-tumor therapy and a crucial indicator for judging tumor prognosis.

**Table 3:** Summary of ubiquitination enzyme regulation of targeting proteins.

Enzyme	Name	Targets	Cancer	Animal models	Reference
E3 ligase	TRAF4	NOX2/NOX4	Non-small-cell lung cancer	Mice	130
	A20	TGF-β	Basal-like breast cancer	Mice	131
DUB	USP27x	Snail	Invasive basal-like breast cancer cells	Mice	129
DUB	USP18	ATGL	Lung cancer cells	Mice	136
DUB	UCH-L1	COL1A1	Uterine leiomyoma		141
		COL3A1		/	
DUB	USP3	COL6A5 COL9A3	Gastric cancer	/	140
E3 ligase	HRD1	MMP2/9	Colon cancer	/	153
	MDM2	MMP9	Metastatic breast cancer	/	151
	TRIM13	MMP9	Clear-cell renal cell carcinoma	/	152
	FBXW2	MMP2/9	Lung cancer	/	146
	UCH-L1	MMP1	Brain glioma	/	145
DUB	OTUD7B	TRAF3	Lung cancer	Mice	154
	USP15	MMP3	Non-small cell lung cancer	Mice	149

### Ubiquitination mediates adipose cell function in the TME

Previous reports have only reported the link between adipocytes and obese patients, but new research has shown that some biomarkers in the adipose tissue of cancer patients can serve as an indicator of cancer characteristics, thereby suggesting a crucial link between tumor cells and adipocytes.^134^ Although a substantial gap exists in the study of the role of ubiquitination in the interaction between adipocytes and tumor cells, the regulatory role of ubiquitination in lipid metabolism is clear. Ubiquitin-specific proteases, such as USP18, can promote the growth of lung cancer cells by inhibiting the degradation of adipose triglyceride lipase and promoting lipolysis and fatty acid oxidation^135^ (Table 3). Targeting ubiquitination to regulate adipocytes and lipid metabolism and ultimately exert anti-tumor effects may be the future research direction.

### Ubiquitination regulates the extracellular matrix of the TME

**Figure 5:**
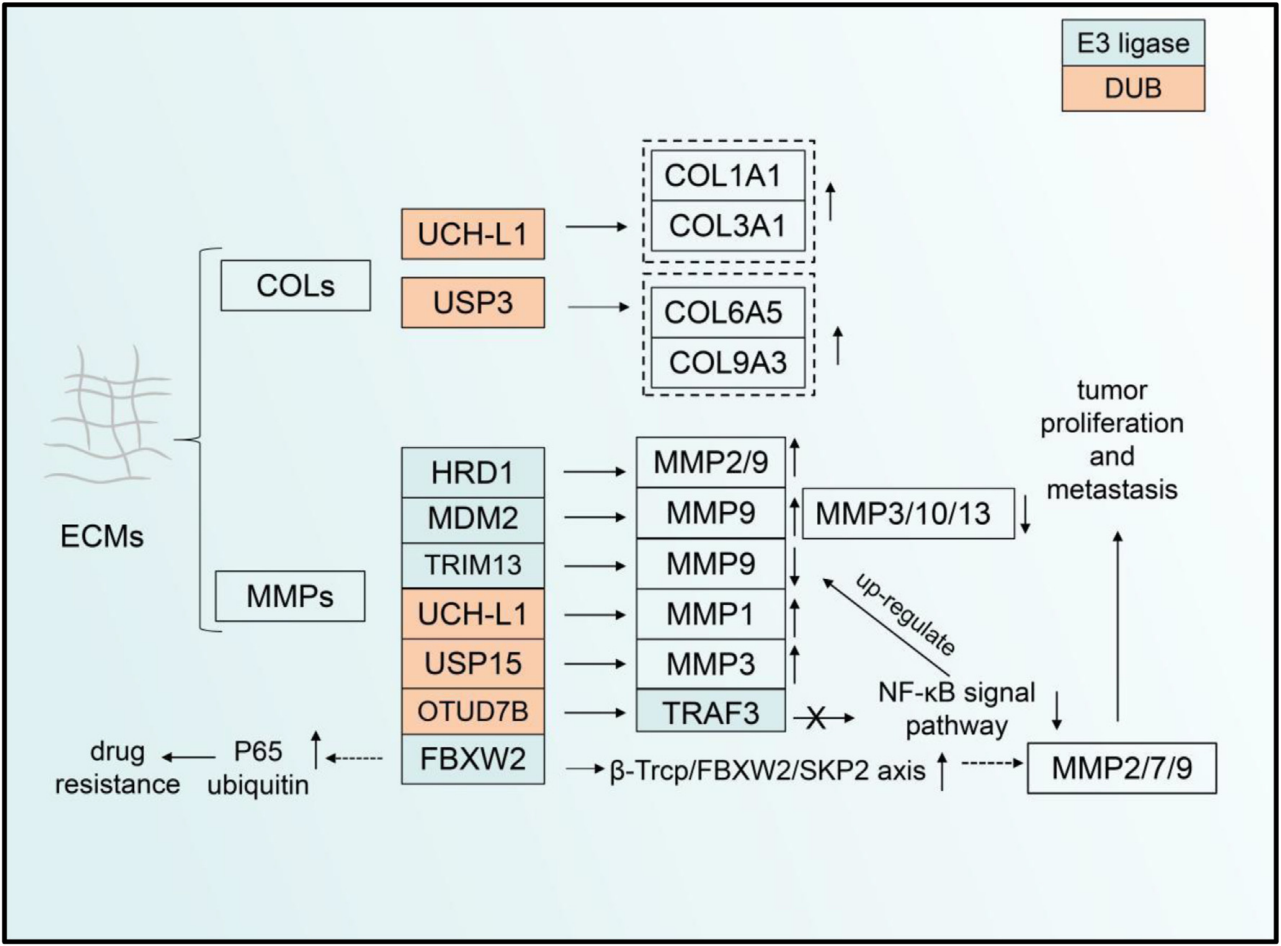
The UPS regulates the extracellular matrix in the TME. COLs and MMPs metalloproteinase are important proteins in ECM, which are regulated by ubiquitin enzymes during cancer progression. The level of COLs and MMPs can be regulated by UPS such as HCHL-L1, USP3, and mdm2. FBXW2 can regulate MMP2/9 protein stabilization through β-Trcp/FBXW2/SKP2 signaling and promote tumor cell proliferation. FBXW2 can also cause drug resistance during clinical treatment by modifying the ubiquitination of P65 protein. ECM, extracellular matrix; COL, collagen; MMP, matrix metalloproteinase; UCH, ubiquitin carboxy-terminal hydrolases; USP, ubiquitin-specific protease; OTU, ovarian tumor proteases; FBXW, F-Box and WD repeat domain containing; TRIM, tripartite motif-containing; SKP, S-phase kinase-associated protein; TRAF, tumor necrosis factor receptor-associated factor; SKP, S-phase kinase-associated protein.

Because the extracellular matrix (ECM) is rich in proteins such as collagens, matrix metalloproteins, and fibronectin. It maintains the overall environmental stability of the TME.^136^ Some ubiquitin proteins can promote tumor proliferation by regulating the protein stability in the ECM and building a “highway” for the rapid migration of tumor cells. Collagen (COL) is the largest protein family in the ECM. As a major component involved in maintaining the ECM framework, collagen is a key player in maintaining ECM stability.^137^ Most current studies on collagen have targeted cell fibrosis, but a few studies have reported ubiquitination-mediated regulation of collagen that promotes tumor cell migration.^138^ COL9A3 and COL6A5 are members of the collagen family (Fig. 5). The deubiquitination enzyme USP3, an essential mediator regulating oncogenic activity both *in vitro* and *in vivo*, can deubiquitinate COL9A3 and COL6A5 in gastric cancer cells. The elevated USP3 expression can affect the abundance of COL9A3 and COL6A5, thereby promoting tumor proliferation and migration of gastric cancer cells.^139^ Moreover, according to a new report, UCH-L1 regulates cancer cell migration and contraction by regulating the stability of COL1A1 and COL3A1 proteins.^140^

The metalloproteinase family also occupies a large proportion of the ECM. This protease family can hydrolyze most proteins in the ECM, and even some cytokines and chemokines, thereby promoting tumor cell growth.^141^^,^^142^ E3 ubiquitinase-regulated matrix metalloproteinases (MMPs) are found in most cancers. For example, RING E3 ubiquitin ligase and HECT ubiquitin ligase are involved in regulating MMP stability and thus affect tumor development.^143^ One report for the first time identified MMP-1, UCHL1, and the 20s proteasome in patient plasma as markers for glioma. However, it could not clarify the specific regulatory relationship among MMP-1, UCHL1, and the 20s proteasome^144^ (Fig. 5). FBXW2, a RING E3 ubiquitin ligase, serves as a vital regulator in lung cancer. FBXW2 promotes MMP2, MMP7, and MMP9 expression by forming the β-Trcp/FBXW2/SKP2 axis with other ubiquitin ligases such as β-Trcp and SKP2.^145^, ^146^, ^147^ The latest report proposes that FBXW2 overexpression in breast cancer leads to p65 ubiquitination, eliminating the effect of p65 resistance on paclitaxel use.^146^ In other tumors such as non-small cell lung cancer, USP15 has been reported to be positively associated with MMP3.^148^ In addition to regulating p53 protein stability, MDM2 also regulates MMP9 protein stability in ECM. There is an association between MDM2 expression in prostate cancer and the expression of MMP family proteins, especially MMP9, which promotes tumor cell migration by balancing pro-angiogenic mechanisms.^149^ Moreover, MDM2 has also been shown to down-regulate the abundance of MMP3, MMP10, and MMP13, with a role in inhibiting the invasion of breast cancer cells.^150^ TRIM13 can inhibit clear-cell renal cell carcinoma invasion by down-regulating MMP9 expression.^151^ HRD1 promotes the proliferation and migration of colon cancer. The expression of this ubiquitin ligase was found to be higher in cancer cells than in other cells, and the expression of MMP2 and MMP9 was also elevated. However, the specific mechanism of how HRD1 regulates MMP2 and MMP9 is still unclear.^152^ In addition, in lung cancer cells, LCL161 drugs could up-regulate the expression of MMP9 protein and thus induce cancer cell migration. OTUD7B inhibits the activation of NF-κ B by deubiquitinating TRAF3, which in turn promotes the transcription of MMP9, thereby exerting an inhibitory effect on the migration of lung cancer cells.^153^

### Prospect of ubiquitination-targeted drugs in tumor therapy

Although many inhibitors regulating ubiquitination have been screened out, very few drugs are truly applied for clinical therapeutic usage. MG132, a modified version of the first proteasome inhibitor, was widely investigated in most laboratories for proteasome inhibition.^154^ Other proteasome inhibitors, such as bortezomib, carfilzomib, and ixazomib, were successively developed. They received FDA approval for clinical treatment, where the drugs exhibited good results in the treatment of various malignant tumors, especially multiple myelomas.^155^ Bortezomib, the first proteasome inhibitor discovered, was developed and exploited in the clinical treatment of multiple solid tumors and hematology tumors. This inhibitor blocks the proteolytic function of the 26S proteasome complex by covalently binding to the β5 subunit of the 20s proteasome.^156^ Clinically, bortezomib can be used alone or in combination with other chemotherapeutic drugs. For example, in the multiple myeloma clinical phase 2 experimental report, complete response/stringent complete response rate improved after treatment with the bortezomib-cyclophosphamide-dexamethasone combination.^157^ The poor solubility of bortezomib owing to its chemical structure makes the clinical translation of this inhibitor difficult despite its excellent therapeutic efficacy. Second, due to the strong toxicity of bortezomib, patients experienced vomiting, nausea, poor mental state, and even abnormal perception symptoms during clinical trials.^158^, ^159^, ^160^, ^161^ Finally, the inhibitor may also lead to drug resistance because of the binding of bortezomib to the β5 subunit of the 20s proteasome, which thus inhibits the binding of the β5 subunit to other subunits.^162^ Therefore, the development of relevant inhibitors based on bortezomib may be improved in future drug development. Subsequently, carfilzomib and ixazomib were also developed in 2012 and 2015, which were used to solve the problem of drug resistance arising during medication. Clinical data after the use of related inhibitors have also been reported.^163^

Of note, E3 ubiquitin ligase is among the most crucial components of the UPS system, which guarantees the highly specific degradation of substrate proteins. Developing inhibitors targeting this ligase can maximize the drug's function. For example, MDM2-targeting-related inhibitors have been developed to block the binding of the MDM2 N-terminal domain to the peptide segment of p53.^164^ Nutlin-3a and its derivatives play pivotal roles in inhibiting the growth of hematological malignancies, glioblastoma, and acute myelocytic leukemia cells because their structure is similar to that of p53 and allows competitive binding of MDM2 to p53.^165^^,^^166^ Other inhibitors targeting MDM2 such as AMG-232 (KRT-232), APG-115, and Brigimadlin have also been reported recently.^167^, ^168^, ^169^ PROTAC is used as a targeted UPS technology for regulating target protein degradation. The mechanism of this technology is not directly targeting E3 ubiquitin ligase, but by recruiting E3 ligase, one end connects to the target protein and the other end connects to E3 ubiquitin ligase, forming a ternary complex of target protein PROTAC-E3ligase, thereby achieving the degradation of the target protein.^170^ This technology has the advantage of reducing drug resistance and toxicity.^171^ It has good effects in treating various cancers. For example, in the treatment of triple-negative breast cancer, PROTAC targeting the MDM2-p53 axis can significantly improve the survival period of tumor-bearing mice.^172^ Although multiple E3 ubiquitin ligases have been discovered, few ubiquitin ligases are targeted by PTROTAC. Such molecules only target classical proteins such as VHL and MDM2,^173^ which means that there are still limitations in tumor treatment. We look forward to developing more types of ligases targeted by PROTAC in the future.

The development of DUB inhibitors is another important target for cancer therapy. Some inhibitors are widespread and can target multiple types of DUB. For example, B-AP15 as a DUB inhibitor can address the problem of resistance arising during bortezomib treatment. It binds to the 26s proteasome to inhibit the function of the deubiquitinating enzymes USP14 and UCHL5.^174^ Another inhibitor, VXL1570, also inhibits the functions of USP14 and UCHL5, which when used alone caused tumor reduction in Waldenstrom's macroglobulinemia tumor-bearing mice. Both the aforementioned inhibitors combined with bortezomib or ibrutinib could kill Waldenstrom's macroglobulinemia cancer cells. Because of the difference in the chemical structures of the two inhibitors, the water solubility of VXL1570 was better than that of BAP15, which resulted in a higher stability of VXL1570 in the patient's body. VXL1570 is approved for use in clinical trials. However, two patients with multiple myeloma developed severe exhalation insufficiency and diffuse pulmonary infiltration due to the severe toxicity and side effects of VXL1570. Thus, the clinical experiment was stopped when the patients died during phase I treatment despite the advantages of a broad spectrum of the inhibitors.^175^ The development of high-specificity of inhibitors is the focus in tumors.^176^

## Conclusions

In the past few decades, the regulatory role of UPS in tumor progression has been extensively studied, especially to determine its impact on the biological behavior of tumor cells themselves and the shaping of the tumor immune microenvironment by tumor cells. Here, we retrospect the regulatory effects of USP on tumor cells, immune cells, stromal cells, and ECMs. This enhances our understanding of ubiquitination and provides a basis for further research on tumor occurrence and development and the development of ubiquitination-targeting anti-tumor drugs. In the study of UPS and tumors, numerous studies have reported the important role played by UPS in tumor cells, T cells, and tumor-related macrophages. However, most current research is limited to the effect of UPS on one cell type. The TME contains multiple cells, which often results in unpredictable other effects in the organism. Only considering its regulatory effects on one or more cells when developing targeted UPS drugs is not appropriate as other unpredictable effects are often observed during clinical treatment. UPS regulates multiple components of tumors and ultimately affects tumor progression. It regulates the cycle, energy metabolism, and protein molecule expression of tumor cells by regulating the ubiquitination and deubiquitination of target proteins. It also regulates the interaction between tumor cells and other cells as well as the function of immune cells and interstitial cells other than immune cells. However, a close synergistic relationship exists between ubiquitination regulation and other post-translational modifications.^80^ Other post-translational modifications may play a regulatory role in protein ubiquitination. Moreover, the protein ubiquitination level can affect other post-translational modification processes. This suggests that attention must be paid to this point in future research. UPS-regulated targeting protein stability reported in some studies is only limited to changes in the protein level, but the specific mechanism remains unclear. Based on the characteristics of the UPS system, the development of related inhibitors such as PROTAC has become the recent research focus.^44^ This type of inhibitor can hydrolyze proteins with the help of the UPS system, which causes the pathological protein to be tagged with ubiquitination, thereby achieving target protein degradation and tumor treatment. However, toxicity- and specificity-related concerns of these inhibitors need to be solved. In addition, this article only describes ubiquitination- and deubiquitination-associated enzymes. Some proteases also possess the function of ubiquitination modification. Such a modification is called ubiquitination-like modification. This type of modification also plays a pivotal role in regulating tumor development and needs to be explored.

## Author contributions

Yulan Huang and Yuan Gao conceived the idea of the manuscript and wrote it. Zhenghong Lin and Hongming Miao revised the manuscript. All authors read and approved the final manuscript.

## Funding

This work was supported in part by the 10.13039/501100001809National Natural Science Foundation of China (No. 82173134) and the Chongqing Fund for Outstanding Youth (China) (No. CSTB2022NSCO-JOX0010).

## Conflict of interests

All authors declared no conflict of interests.
